# Technological Challenges in the Development of Optogenetic Closed-Loop Therapy Approaches in Epilepsy and Related Network Disorders of the Brain

**DOI:** 10.3390/mi12010038

**Published:** 2020-12-31

**Authors:** Bram Vandekerckhove, Jeroen Missinne, Kristl Vonck, Pieter Bauwens, Rik Verplancke, Paul Boon, Robrecht Raedt, Jan Vanfleteren

**Affiliations:** 1 Center for Microsystems Technology, Imec and Ghent University, 9000 Ghent, Belgium; Bram.Vandekerckhove@UGent.be (B.V.); Jeroen.Missinne@UGent.be (J.M.); Pieter.Bauwens@UGent.be (P.B.); Rik.Verplancke@UGent.be (R.V.); 2 4Brain Team, Department of Head and Skin, Ghent University, 9000 Ghent, Belgium; Kristl.Vonck@UGent.be (K.V.); Paul.Boon@UGent.be (P.B.); Robrecht.Raedt@UGent.be (R.R.)

**Keywords:** optogenetics, optrode, brain, epilepsy, biocompatibility, closed-loop therapy, flexible implants

## Abstract

Epilepsy is a chronic, neurological disorder affecting millions of people every year. The current available pharmacological and surgical treatments are lacking in overall efficacy and cause side-effects like cognitive impairment, depression, tremor, abnormal liver and kidney function. In recent years, the application of optogenetic implants have shown promise to target aberrant neuronal circuits in epilepsy with the advantage of both high spatial and temporal resolution and high cell-specificity, a feature that could tackle both the efficacy and side-effect problems in epilepsy treatment. Optrodes consist of electrodes to record local field potentials and an optical component to modulate neurons via activation of opsin expressed by these neurons. The goal of optogenetics in epilepsy is to interrupt seizure activity in its earliest state, providing a so-called closed-loop therapeutic intervention. The chronic implantation in vivo poses specific demands for the engineering of therapeutic optrodes. Enzymatic degradation and glial encapsulation of implants may compromise long-term recording and sufficient illumination of the opsin-expressing neural tissue. Engineering efforts for optimal optrode design have to be directed towards limitation of the foreign body reaction by reducing the implant’s elastic modulus and overall size, while still providing stable long-term recording and large-area illumination, and guaranteeing successful intracerebral implantation. This paper presents an overview of the challenges and recent advances in the field of electrode design, neural-tissue illumination, and neural-probe implantation, with the goal of identifying a suitable candidate to be incorporated in a therapeutic approach for long-term treatment of epilepsy patients.

## 1. Background

Epilepsy is a chronic brain disease characterized by the occurrence of epileptic seizures. An epileptic seizure is a transient occurrence of signs and/or symptoms due to abnormal excessive or synchronous neuronal activity in the brain [1]. Epilepsy carries neurological, cognitive, psychological and social consequences and accounts for a significant proportion of the world’s burden of disease, affecting around 50 million people worldwide [2]. Epilepsy is typically treated with anti-epileptic drugs (AEDs). About one third of patients suffer from drug-resistant epilepsy (DRE) and keep having seizures despite trying several AED treatment regiments [3]. The leading cause for treatment failure with AEDs are adverse effects. Not only do they result in early treatment discontinuation in up to 25% of patients, but they also preclude attainment of fully effective doses and have a negative effect on patient adherence. Furthermore, adverse effect of AEDs are a major source of disability, morbidity, and mortality and a substantial burden on use and costs of healthcare [4]. 

Non-pharmacological treatment strategies for epilepsy are available such as ketogenic diet [5], vagus nerve stimulation [6], deep brain stimulation [7] and resective surgery [8]. There is also a continuing search for better treatments with higher spatial and temporal specificity, which target only epileptic brain regions at times when risk for seizures is high. Closed-loop strategies that combine brain recordings of epileptic activity and therapeutic strategies that decrease hyperexcitable tissue may provide a valuable alternative [9].

Optogenetics allows modulating neuronal activity, with an unprecedented spatiotemporal resolution and cellular specificity. This technique consists of inducing cellular expression of light sensitive protein (i.e., opsins) and subsequent modulation of cellular activity with light. These opsins are typically ion channels, ion pumps or receptors derived from type 1 microbial opsins. The best known opsins are channelrhodopsin 2 (ChR2) [10], which is a blue-light sensitive cation channel used for neuronal excitation [11], and halorhodopsin (NpHR), which is a yellow-light sensitive chloride pump resulting in neuronal inhibition [12]. However, currently researchers have access to a large toolbox of excitatory and inhibitory opsins with different channel kinetics and wavelength sensitivities [13,14].

Although optogenetics was initially developed as a research technique, it has become clear in recent years that this neuromodulation technique could be further developed towards a clinical therapy. Optogenetics seems to be especially well suited for closed-loop interventions in epilepsy, where seizure activity is interrupted in its earliest state by light-induced activation of opsins [15,16,17]. Closed-loop optogenetics requires the use of optrodes, which consist of a recording electrode to detect early seizure onset and an optical component to provide sufficient light power for local activation of opsins in the seizure focus. Although the minimal amount of tissue that needs to be optogenetically modulated to stop seizures is unknown, optogenetic interventions in different parts of the epileptic network can be equally effective in interrupting seizure activity. Temporal lobe seizures for example can be successfully interrupted by modulation of different temporal lobe brain regions, such as hippocampus and dentate gyrus, as well as brain regions connected to the temporal lobe, such as the superior colliculus and cerebellum [17,18,19,20,21,22,23,24]. Two optogenetic approaches successfully stop seizures: selective inhibition of excitatory neurons by activation of inhibitory opsins or selective excitation of inhibitory neurons by activation of excitatory opsins.

Different types of epilepsy are associated with epileptic activity in different brain networks and thus will require different optrode designs for recording and optical stimulation of different brain regions. Additionally, since epilepsy is a chronic disorder these optrodes should maintain their recording/illumination functionality for years up to decades after implantation. Therefore, this review discusses both electrical and optical probes, with the aim of identifying suitable designs that can provide the required recording and stimulation capabilities, without inducing detrimental biological responses that could compromise the optrode’s long-term functional longevity.

## 2. Biological Constraints of Long-Term Implantation of Optrodes

Neural implants can lose their functionality after weeks post-implantation as a result of the so-called foreign body reaction (FBR) [25,26,27]. This reaction is defined as the end-stage condition of an inflammatory and wound healing response following implantation of a medical device, prosthesis, or biomaterial. Histologically, the FBR is characterized by a sequence of events evolving from acute to chronic inflammation and primarily consisting of foreign body giant cells consisting of fused monocytes and macrophages. These are activated during the acute inflammatory state immediately following blood-brain barrier (BBB) injury when a foreign body is introduced to brain tissue. In parallel with the foreign body giant cell formation, granulation tissue is formed leading to a fibrous capsule around the implanted device. Due to the BBB injury and the persistent presence of the implanted probe, gliosis expresses itself in multiple ways [28]. Shortly after the insertion, the amount of activated microglia increases significantly in the proximity of the probe [29]. These cells start excreting degenerative proinflammatory factors, interleukins, in an attempt to remove the intruding object [28]. Next, activated astrocytes gather around the implant to compose a dense glial sheet to physically isolate the probe from the surrounding neural tissue. The corresponding scar tissue has been shown to completely encapsulate implanted probes and was in some occasions even able to (partially) push implanted electrodes out of the neural tissue [25,30]. Neuronal apoptosis as a consequence of the degenerative factors [29] and the dense encapsulation layer, can compromise the electrical and optical functionalities of implants. Therefore, design considerations for chronic implants should focus on the reduction of the various aspects of this FBR.

The FBR is partly mediated by the mechanical mismatch between the neural tissue and the probe [31,32,33,34,35,36]. This mechanical mismatch is usually indicated by the difference in their respective Young’s modulus, a mechanical property denoted by E. The lower the Young’s modulus of a material, the larger the linear elastic deformation as a result of an axially, applied stress. The Young’s modulus of silicon, a material that is used a lot for intracortical probes is 130–190 GPa [37], while neural tissue has a modulus around 2 kPa [38]. Micromotions caused by a combination of the high-frequency vascular and low-frequency respiratory pulsations [39], and the brain’s movement due to body’s motion cause persistent irritation of neural tissue surrounding an implanted probe. Finite-element simulations indicate that lowering the Young’s modulus of the implanted probes can significantly reduce the mechanical strains on the surrounding neural tissue as a result of these micromotions [40] and, therefore, potentially reduce the FBR. Indeed various studies indicate that reducing the implant’s Young’s modulus results in less reactive astrocytes/microglia, a thinner glial sheet surrounding the probe, less chronic BBB leakage and reduced neuronal loss near the probe [31,32,33,34,35,36]. Several studies further indicate that limiting the cross-sectional area of the implant, which reduces the implantation injury and neural tissue exposed to the probe, also reduces the FBR [34,41,42].

## 3. Neural Activity Monitoring

A neural implant for optogenetic interventions needs to include extracellular recording electrodes to detect the aberrant electrophysiological activity associated with the onset of epileptic seizures [1]. These electrodes are capable of recording both local field potentials as well as action potentials of individual neurons and a large variety have been presented. For example, there are the wire/microwire-based electrodes in which every wire corresponds with a single recording site. Usually, these systems contain a single [43,44] or a few (micro)wires [45,46,47,48], although microwire probes containing dozens [49,50] up to hundreds [51] of microwires have been reported. Additionally, there are also the Utah arrays [25,52], silicon-based microelectrode arrays which resemble a ‘bed of nails’. Similar to the microwire type, every ‘nail’ corresponds to a single recording site, although due to the array’s 3D-character the recordings sites are spaced further apart. A last example of extracellular recording probes are the Michigan-type multielectrode arrays. In contrast to the previous types, these silicon-based probes contain multiple electrical recording sites on a single shank, with the maximum number of sites usually determined by the amount of independent signal traces that fit on the probe. Therefore, most of these probes only contain 4 to 8 recording sites per shank [53,54,55]. However, recent advances in silicon processing enable much higher recording site densities and as such thin silicon probes with hundreds of recording sites per shanks have been reported [56,57]. For example, the Neuropixels 1.0 and 2.0 contain respectively 966 and 1280 recording sites per shank (70 µm x 20 µm), of which 384 can be read out simultaneously [58,59,60]. Additionally, due to their high electrode density, they have shown to be capable of recording and tracking hundreds of individual neurons for multiple weeks. Therefore, at this point in time, the Neuropixels probes are the most advanced probes for extracellular recording and the gold-standard in acute and chronic electrophysiological experiments [61,62,63].

The spatiotemporal dynamics of neuronal activity underlying the generation of epileptic seizures are still incompletely understood. Local field potentials in the affected neural area can indicate seizure activity, but detection of the neuronal activity (i.e., action potentials) driving the seizure activity has proven to be extremely difficult [64]. Traditionally seizures have been detected and studied with electroencephalogram (EEG) electrodes placed on the patient’s scalp, but since a few years the use of some microelectrode arrays have been approved and this has unraveled a clear distinction between seizure dynamics on the macroscopic and microscopic level. While EEGs can detect abnormal patterns over large brain areas during seizures, the expected hypersynchronized neuronal firing patterns are only detected on a limited number of microelectrodes and appear to spread like a wave over the tissue (ictal wave). Due to the field’s uncertainty regarding the seizure generation mechanism, no consensus on the number and density of recording sites for early onset detection has been obtained. Nevertheless, since epileptic foci are difficult to pin-point, electrical recording probes with several recording sites spatially distributed over an extended volume are hugely preferred compared to single macroelectrodes. Consequently, 3D microelectrode arrays such as presented by Kim et al. [65] and Chung et al. [66], are interesting for this application. Furthermore, a high signal-to-noise ratio (SNR) of the recorded signals is a prerequisite [67]. A high SNR requires the recording electrodes to be in close proximity to the signal origin (i.e., firing neurons) and to exhibit a low electrode-tissue impedance. Both requirements can be compromised when the implant causes a severe FBR, which causes neuronal loss in the proximity of the probe [31,32,33,34] and increases the electrical impedance over time due to gliotic scarring [68].

It can be concluded that the technical requirements for epilepsy detection (both in humans and rodents) come down to the following three points. Firstly, the probe may only elicit a minimal foreign body response. Secondly, a low electrode-tissue impedance (in the order of tens of kiloohms) is desirable, since it increases the SNR. Thirdly, depending on the selected seizure detection method, the neural probe may require spatially distributed recording sites, such that the neural activity can be measured over a sufficiently large volume.

### 3.1. Electrode Designs That Elicit a Minimal Foreign Body Response

**Figure 1:**
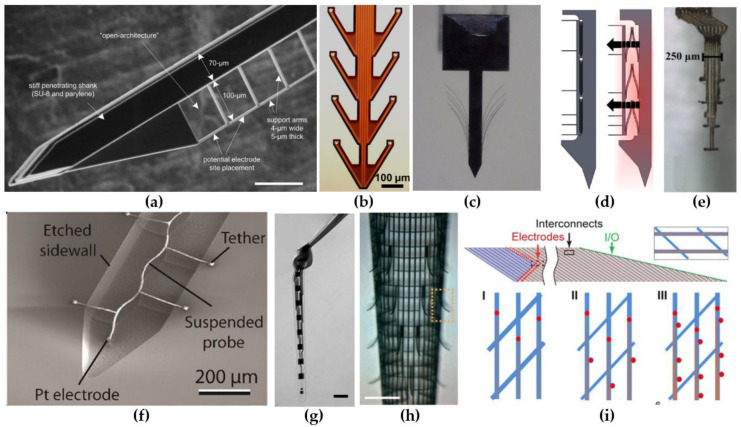
Electrode designs for reduced foreign body reaction (FBR). (**a**) An intracortical probe with a thin, lateral platform, which allows positioning of the recording sites 100 µm away from the main, perturbating probe shank. Scale bar, 100 µm. Reproduced with permission from [42]; published by Elsevier, 2007. (**b**) A fish-bone shaped neural probe. Reproduced with permission from [69]; published by IEEE, 2011. (**c**) Photograph of the splaying probe in agar after slightly retracting. Reproduced with permission from [70]; published by IEEE, 2019. (**d**) Illustration depicting the state of the self-deploying probe before and after the dissolution of the temporary glue. Reproduced with permission from [71]; published by IEEE, 2011. (**e**) A highly-flexible electrode array consisting out of eight encapsulated gold leads. Each lead is able to move independently from the others, since they are not interconnected. Reprinted with permission from [72]; published by Frontiers, 2015. (**f**) Ultra-miniature, meandered probe, with a single Pt electrode at the end. Reproduced with permission from [73]; published by Springer, 2016. (**g**) A NET-50 probe suspended in water, knotted to illustrate the ultra-high flexibility. Scale bar, 50 µm. Reprinted with permission from [74]; published by AAAS, 2017. (**h**) A cylindrical, mesh electrode with outward-bending arms containing the electrical recording sites. Scale bar, 200 µm. Reproduced with permission from [75]; published by Nature, 2015. (**i**) A planar mesh electrode containing 1, 2 and 4 recording sites, respectively, on each longitudinal element. Reprinted with permission from [76]; published by PNAS, 2017.

A first design strategy consists of using a probe where the electrode contacts extend several micrometers away from the main shank on thin flexible carriers (Figure 1a–d). This strategy aims at positioning the recording contact outside the regions where neuronal loss and inflammatory response due to probe insertion is highest. The benefits of this strategy have been confirmed by Seymour and Kipke [42]. They attached a thin lateral platform, with a cross-section of 4 µm × 5 µm, 100 µm away from the thicker (48 µm × 68 µm) penetrating shank (Figure 1a). Four weeks post-implantation, the neuronal loss around the thin platform edge was one-third lower than at the corresponding region of the thick shank. In addition, the amount of activated microglia and extracellular protein deposition was reduced in close proximity to the recording sites. Wu et al. [69] developed a flexible, fish-bone-shaped, neural probe according to the same principles (Figure 1b). The fish-bone geometry allows the recording electrode contacts to be positioned, on thin side-arms (cross-section of 14 µm × 10 µm), 100 µm away from the central shank, while the substrate-free regions support close integration with the neural tissue. A third concept was demonstrated by Massey et al. [70], who developed a “splaying” probe, with small compliant whiskers (3 µm × 5 µm),which can be spread by slightly retracting the probe (Figure 1c). Alternatively, the recording sites can also be pushed away from the main shank by employing self-deploying satellite sites [71] (Figure 1d). The satellite sites are located on microsprings which are originally fixated with dissolvable glue, but are released after the glue dissolves in the cerebrospinal fluid (CSF).

A second design strategy focused on creating ultra-flexible probes with a minimized cross-sectional area. Small, ultra-flexible probes have been manufactured with polyimide (E = 2.5 GPa [77]), parylene-C (E = 2.8 GPa [78]) and SU-8 (E = 3 GPa [79]). For example, Musk and Neurolink developed and fabricated more than 20 polyimide-based thread and electrode types with widths varying between 5 and 50 µm and a thickness of 4–6 µm [80]. Furthermore, Agorelius et al. [72] presented a microelectrode array with eight individually flexible leads (Figure 1e). Each lead consisted out of a gold wires, sandwiched between two 4-µm-thick parylene-C insulation layers, resulting in a cross-sectional area for each lead of only 150 µm^2^. An even more miniaturized (cross sectional area of 27 µm^2^) parylene-C-based neural recording probe was demonstrated by Khilwani et al. [73]. Their probe consisted out of insulated, meandered Pt wire connected to a 26-µm-diameter disk shaped electrode (Figure 1f). The miniature probe size makes the probe ultra-compliant along the radial direction, while the meandered geometry provides an improved flexibility along the axial direction [73,81]. Another type of ultra-flexible neural probes are the nanoelectronic thread (NET) brain probes originally proposed by Luan et al. [74,82] (Figure 1g). The NET probes use a substrate-less, multilayer architecture to achieve unprecedented thin probes with a total thickness equal or less than 1.5 µm. Multiple different designs have been presented. Firstly, their four-layer NET-50 probe (Figure 1g) was based on the well-known silicon microelectrode probes. The probe hosts eight linearly positioned electrodes along the 50 µm x 1 µm shaft. Secondly, the NET-10 probe is a seven-layered neural probe providing two electrodes on both sides. At the time that the authors presented the NET-10 probe, they believed it was the smallest among all reported neural probes due to its cross-sectional area of only 15 µm^2^ (10 µm × 1.5 µm). One year later, they outperformed themselves by presenting their NET-e series, neural probes with a cross-section of 8 µm × 0.8–1 µm [82]. While these probes are even a smaller than the NET-10 probe, they showed that complex multielectrode configurations are still viable. They did this by presenting a linear, a tetrode-based and an oversampling-based probe, containing 8, 16 and 16 electrodes respectively. Further, they have proven the long-term reliability of the NET probes by implanting 16 NET-50 probes into the somatosensory and visual cortices of seven mice for four months. They noted that the average impedance and observed noise level decreased for the first 1.5 months and then remained stable for the next 2.5 months. Similarly, the amount of detectable unit events and sortable single-unit action potentials increased over the first 1.5 months and remained stable for the rest of the experiment. Additionally, histological staining 3.5 months post-implantation showed no observable chronic tissue reaction at the interface. All this suggests that NET-probes show an almost unprecedented biocompatibility and are capable of long-term stable recording of LFPs and single-unit action potentials.

A last type of ultra-flexible electrodes are the mesh electrodes. Mesh electrodes have been proposed as part of flexible probes before [37], but no exceptional results were obtained. More recently, Xie et al. [75] presented their free-standing, three-dimensional, macroporous, nanoelectronic networks (Figure 1h). This mesh network is composed out of parallel, SU-8 coated longitudinal, metal interconnects, transversally connected with compressive strain elements that provide the mesh its cylindrical shape. Besides the compressive transversal elements, the probe also contains 19 tensile strain elements, that curve away from the mesh structure, on which the electrodes are located. Moreover, the total design has a two-dimensional open area of approximately 80% and all elements have feature sizes below 10 µm. This causes the mesh electrode to achieve a very high flexibility, with an effective bending stiffness, which, as the authors report, is four to seven orders of magnitude smaller than most silicon, carbon fiber and thin polyimide neural probes. Further, the probe exhibits very good biocompatibility, since five weeks post-implantation, the neural density in close proximity to the probe (<50 µm) showed normal neural growth. The original void caused by the roughly cylindrical mesh probe also got filled with cells and/or neural projections, indicating the interpenetration of cells through the macroporous structure. Similar results were obtained for the mesh probe presented by Zhou et al. [83,84]. They reported that three months past implantation the amount of neuronal nuclei, neurofilaments, activated microglia and astrocytes inside and at the surface of the probe were the same as for tissue located far away. This suggests that highly flexible mesh electrodes elicit no chronic biological reaction and, therefore, are just as NET-probes, ideally suited for long-term implantation. Further, the amount of recording sites on the mesh electrodes were originally quite limited, since each longitudinal element only contained a single recording site. To cope with this limitation, Zhou et al. [83] improved on their design by demonstrating the viability of incorporating up to four recording sites on each longitudinal element (Figure 1i), while still maintaining the probe’s flexibility in the same range as the flexibility of neural tissue [76].

### 3.2. Reduction of the Electrode-Tissue Impedance

Electrode contacts of intracerebral microelectrodes are often fabricated out of Au, Pt or Ir [85,86,87], due to their biological inertness and biocompatibility. The metal contacts can record neural activity by converting the electrolytic-based currents in the biological environment to electron-based currents in the metal. There are two types of processes that allow this conversion [67,88]. The first one is called capacitive charge injection and employs the electrical double layer (EDL) at the electrode-tissue interface, which can be envisioned as a capacitor. When a neuron depolarizes or fires, positive ions flow into the cell and negative ions move away from the neuron to maintain the charge neutrality in the extracellular medium. The ions arriving at the EDL cause an accumulation of negative electrolytic-based charge at the interface, which causes electrons inside the metal to propagate towards the detector. The second process is known as the Faradaic charge injection and employs electrochemical reactions, such as oxidation and reduction, at the electrode-tissue interface to transport electrons from the extracellular medium into the metal or vice versa. In the equivalent circuit model of the electrode-tissue interface, the Faradaic currents can be envisioned as due to a resistor in parallel with the EDL’s capacitor. Consequently, the total impedance of the interface can be decreased by either increasing the EDL’s capacitance or decreasing the resistance, both which are achieved by increasing the area of the metal contacts [67,88]. The most straightforward way to improve the SNR is thus by enlarging the recording electrode. However, as was discussed earlier, this could have detrimental effects on the electrode’s biocompatibility and functional longevity. A better option is to make use of the 3D-character of the probe, to create larger recording sites without actually enlarging the probe. The probe presented by Wu et al. [69] illustrates this concept. They sputtered a supplementary Ti/Ir layer on their Ti/Au contacts and on the sidewalls of the polyimide insulation layer to more than double the size of the electrical contacts. Another option, illustrated by Du et al. [89] and Seymour et al. [79], is to also add electrical recording sites to the bottom and sides of the electrode, which significantly increases the available surface area. 

Alternatively, the effective surface area of the metal contacts can also be increased by increasing the metal’s roughness or creating micro-or nano protrusions on top of the metal contacts. Some of the reported methods involve the deposition of platinum particles [45], iridium oxide [45], platinum black [90], Pt-nanograss [27,91], gold [89] and carbon nano tubes (CNT) [49,92]. Another option is the deposition of conductive polymers, such as poly(3,4-ethylenedioxythiophene) (PEDOT) [82,93,94,95], polypyrrole (PPy) [93] and polyaniline (PAni) [96]. Besides lowering the impedance, these polymers also offer an improved mechanical interface with the surrounding biological medium. In addition, the conductive polymers can be doped with anionic dopants such as poly(styrene sulfonate) (PSS) [97,98,99] and p-toluenesulfonate (pTS) [93] or combined with CNTs [94] to further improve their conductivity. Table 1 lists the quantitative impedance improvements that were reported for each of these methods. 

Noteworthy, conductive polymers can also be altered to contain bioactive molecules, such as dexamethasone and nerve growth factor, inside the polymer networks [45]. The release of the anti-inflammatory dexamethasone after implantation has shown to greatly attenuate the acute astrocytic and microglial response from a few days to more than six weeks, hereby limiting the chronic effect of the initial stab wound [100,101]. Additionally, the nerve growth factor can be used to enhance neuron survival and promote neuron growth toward the microelectrodes [102], hereby, further improving the signal strength and thus the SNR.

## 4. Neural Tissue Illumination

Another crucial factor for designing a functional optogenetic probe is the inclusion of an appropriate illumination tool for the selected optogenetic approach. As mentioned in the introductory section, optogenetic approaches vary regarding the selected opsin, the volume of targeted tissue, the corresponding required light power, frequency and duration of light pulses, and overall duration of the optical stimulation. Consequently, at this point in time no single illumination tool can be selected as the ideal component. Nevertheless, similar to the electrical recording electrodes, this illumination tool should only elicit a minimal FBR.

Activation of opsins for optogenetic modulation of neurons requires illumination with light of a specific wavelength at a minimal, light-power density. The effective light-power density for which 50% of the opsins get activated is called the EPD50. For ChR2 and NpHR the EPD50s lay around 1.3 mW/mm^2^ at a peak activation wavelength of 473 nm and 5.4 mW/mm^2^ at a peak activation wavelength of 589 nm, respectively [103,104]. Nevertheless, EPD50s can range as low as 0.01 mW/mm^2^ for SOUL [105], to as high as 15 mW/mm^2^ for PsChR [104,106]. Ideally, the light power density to modulate opsin-expressing neurons is kept as low as possible across the targeted volume of neural tissue. Unfortunately, this is difficult to obtain, as result of the high absorption (µ_a_) and reduced-scattering (µ_s_’) coefficients that both grey and white matter exhibit. Values of the absorption and reduced-scattering coefficient for 473-nm-light are reported ranging between 0.5–5 and 10–50, respectively [107]. The reduced-scattering coefficient is a variable combining both the absorption coefficient (µ_s_) and the anisotropy (g) of neural tissue according to µ_s_ = µ_s_’/(1−g) [108]. To cope with the complex light-propagation mechanics inside neural tissue, simulation tools, based on 3D Monte Carlo models, are made available [108]. These tools allow the user to estimate the illumination profile and the total volume of activated neural tissue based on the properties of the optical source [108]. Previous papers reported results of simulations to demonstrate the effect of the numerical aperture (NA) and the light source [13,107,109]. The NA and the size of the source showed to have only a limited effect on the illumination profile and on the volume of activated tissue, as a result of the large scattering coefficient of brain tissue. In contrast, both employing light with a longer wavelength and increasing the transmitted optical power, shows to be beneficial for large volume illumination. The absorption and scattering coefficients of neural tissue are largely wavelength dependent and decrease for longer wavelengths [107,108]. Therefore, red-shifted opsins are easier to activate across large volumes than their blue shifted counterparts. Alternatively, large volumes of opsin-expressed neurons can be activated by increasing the irradiated light power and, correspondingly, the light-power density, at the source-tissue interface. However, practically, the light-power output needs to be restricted to prevent an excessive increase of the local temperature. Temperature increases of only 1 °C have shown to affect neuronal function [110,111]. Therefore, designs usually limit the permittable tissue heating to 0.5 °C [112,113], which restricts the amount of neural tissue that can be illuminated with a single source. Thus, a single external light source cannot be used to activate neural tissue located below the cortical surface and, consequently, dedicated light delivery systems have to be implemented. Two possible solutions, based on either implantable microLEDs (µLEDs) or waveguides, are discussed in the sections below. The main difference between the two methods is the way in which they provide the light inside the neural tissue. µLED-based systems bring the light sources inside the tissue and convert electrical current to light in situ. In contrast, waveguide-based systems employ light sources located outside the brain and use light guiding channels to bring the illumination to the desired location, hereby, omitting the need for an active electrical component inside the brain.

### 4.1. Implanted µLED-Based Light Delivery

**Figure 2:**
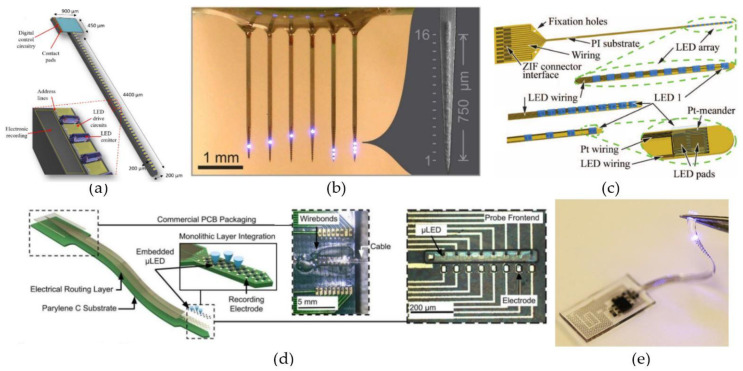
µLED-based neural probes. (**a**) Scalable architecture of the CMOS optrode with 49 LEDs deposited along the 4400-µm-long shaft. Reproduced with permission from [114]; published by IEEE, 2018. (**b**) Multi-shank probe with 96 individually addressable µLEDs distributed along 6 shanks. Reproduced with permission from [112]; published by Nature, 2016. (**c**) Optical cochlear implant constructed by individually flip-chip bonding ten LEDs on top of a 12-µm-thin, highly flexible, polyimide-based probe. Reproduced with permission from [115]; published by IEEE, 2016. (**d**) High density, flexible optrode with embedded gallium nitride µLEDs. Reproduced with permission from [116]; published by Frontiers, 2019. (**e**) Fully implantable, wireless optogenetics system, with a PDMS encapsulated LED, for optogenetic modulation of the spinal cord and peripheral nervous system. Reproduced with permission from [117]; published by Nature, 2015.

The first type of light-delivery system uses µLEDs implemented on carriers to bring the light source(s) in close proximity of the targeted neurons. Zhao et al. [114] presented a µLED-based, optoelectronic probe with integrated CMOS circuits for individual intensity and pulse-width control of 18 µLEDs located along a 4.4-mm-long silicon shaft. Theoretically, 49 individually controlled µLEDs could be placed along the shaft (Figure 2a), or even 88 if more complex routing techniques were used. Upscaling the number of µLEDs on the probe can offer an individually controllable ‘pixel’ size of 90 µm × 90 µm, approximately the size of a single neuron. Further, the integrated CMOS circuits also included a diagnostic functionality, by monitoring impedance changes, hereby providing a verification method of each µLED’s operational status after implantation. Another scalable design for multi-site illumination was proposed by Scharf et al. [112]. They have created a high-density µLED array containing 96 25-µm-diameter individually controllable µLEDs on a silicon substrate (Figure 2b). By using the silicon as a heat sink, they were able to permit a set of optogenetic operating regimes limiting the overall temperature increase to 0.5 °C. Similarly, also sapphire substrates have been shown to provide good enough thermal control for the implementation of multiple 40-µm-diameter GaN-based µLEDs without crossing the 0.5 °C threshold during pulsed operation [113]. 

The properties that make the aforementioned µLED-based systems interesting, are their straightforward scalability [112,114], the high light-source density and the ability to control the radiance profile of each µLED individually [112,113,114]. As a result of those properties, these kind of probes are capable of large volume illumination with high spatial and temporal resolution. Unfortunately, the large mechanical mismatch between the stiff silicon/sapphire substrates and the surrounding tissue make them prone to a severe foreign body response in vivo [35,36]. µLED probes made with more flexible, polymeric materials, such as SU-8 [118], polyimide [115,119,120] and parylene-C [116], have been presented. Originally, these flexible probes could not compete with their stiffer counterparts in terms of either scalability or compactness. The designs of Fan et al. [118], Coa et al. [119] and Schwaerzle et al. [120] all were very bulky (thickness ≥ 350 µm) and only contained a single (µ)LED. Luckily, higher density probes have recently been reported [115,116]. Schwaerzle et al. [115] presented a cochlear implant with ten independently controlled LEDs positioned with a pitch of 350 µm on top of a 12-µm-thick polyimide substrate (Figure 2c). To fabricate the linear LED array, each LED was individually placed with the help of a highly accurate flip-chip bonder. Another approach used GaN-based µLEDs grown on silicon, which were later released and encapsulated with parylene-C, to fabricate a high density, flexible, optoelectronic neural probe [116] (Figure 2d). The probe could achieve µLED densities similar to the densities for the silicon and sapphire probes and designs containing both one- and two-dimensional µLED arrays, incorporating up to 32 µLEDs, were presented. 

Lastly, an even softer µLED-based system was demonstrated by Park et al. [117] (Figure 2e). They presented a fully implantable miniaturized optoelectronic system for wireless optogenetics in the spinal cord and the peripheral nervous system. Both those regions are prone to a lot of movement, so for this reason they developed a µLED-based system able to withstand some straining. By using detached serpentine electrical interconnects and encapsulating the entire structure in polydimethylsiloxane (PDMS), the finished probe had a Young’s modulus of only 1.7 MPa, five orders of magnitude below the modulus of bulk silicon, and could endure strains up to 40%.

### 4.2. Waveguide-Based Light Delivery

The second method of delivering light to deep-located neural structures is with the help of waveguides. Waveguides are passive, optical components than can confine light to its physical boundary as long as the light-guiding core of the waveguide has a higher refractive index (denoted with *n*) than the surrounding material, or “cladding”. As a result, light rays with incident angles within a certain range, determined by the difference in refractive index, will be bounced back at the waveguide edges and therefore remain trapped inside the high-index material. This physical phenomenon is called total internal reflection (TIR) and allows light to be guided along the length of a waveguide. Therefore, optrode designs often implement waveguides, since it allows the designer to position the light source outside of the sensitive neural tissue, whilst still enabling illumination of the modified neurons. 

Noteworthy, the losses of the waveguide systems define, together with the original light power density of the light source, how much light power eventually will arrive at the neurons. There are two important loss contributions in waveguide-based systems. A first contribution involves the coupling losses between the light source and the waveguide. These losses primarily depend on the illumination profile of the light source, the acceptance angle of the waveguide, and the alignment and overlap cross-sectional area between both. The waveguide acceptance angle (2 × alpha) is usually specified by the NA parameter, i.e., NA = sin (alpha) = $\sqrt{n_{core}^{2}-n_{clad}^{2}}$. The larger the waveguide’s NA, the larger the range of angles of light rays (from the source) the waveguide will accept. Similarly, the larger the NA, the wider the cone of light the waveguide will emit onto the tissue. To guarantee a sufficiently high NA, a cladding layer with a well-known, low refractive index is often deposited around the light-guiding core. Without a cladding layer, the NA of the waveguide depends on the refractive index of the surrounding media and it has been shown that this value can vary a lot in neural tissue (*n* ≈ 1.34–1.41 [121]). A second contribution involves the propagation losses which are a measure for the amount of transmitted light inside the light-guiding core. Low propagation losses can be achieved by (i) employing waveguide materials with low optical absorption (at the operation wavelength) and (ii) ensuring a low-roughness boundary between the core and cladding material to limit scattering losses. 

There are two classic waveguide-based approaches that are frequently used to deliver light to deep-located neural tissue. A first approach employs waveguides created on top of stiff (e.g., silicon) carriers with the help of lithography, deposition and etching steps, while a second approach makes use of free-standing optical fibers. Both groups have their own advantages regarding size, stiffness, illumination profiles, losses, etc. More recently, a third group of waveguide-based light delivery systems is introduced. This group is derived from the classical approaches, but employs less conventional biomaterials to construct customized, low-modulus waveguides and optical fibers.

#### 4.2.1. Waveguide-on-Carrier

**Figure 3:**
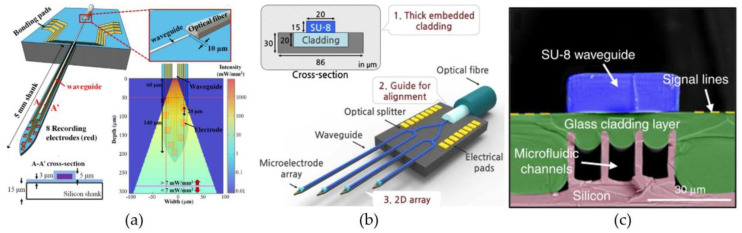
Optrodes constructed with waveguides on Michigan-type neural electrodes. (**a**) An optrode consisting out of an oxynitride waveguide and eight electrical recording sites on top of a 15-µm-thick silicon substrate. The light is coupled into the waveguide by an optical fiber positioned in a U-groove at the proximal end of the probe. For 7 mW of 473 nm light inside the optical fiber, the simulation results show the light-power densities that can be achieved in the neural tissue at the distal end of the waveguide. Reproduced with permission from [122]; published by IOP, 2013. (**b**) A four-shank waveguide-based optrode, constructed using two levels of y-shaped optical splitters. The SU-8 waveguide is accompanied by a low-stress, glass cladding layers created with a reflow process. Reproduced with permission from [87]; published by Nature, 2015. (**c**) Cross-sectional image of the glass cladding layer with incorporated microfluidic channels. Reproduced with permission from [125]; published by Nature, 2019.

The first waveguide-based optical probes that are discussed in this section are the designs that use lithography, deposition and etching steps to fabricate small waveguides on top of silicon probes. These types of probes often also incorporate multiple metal microelectrodes, since they are relatively easy integrated on silicon substrates [87,122,123] (Figure 3a–c). Two similar probes were presented by Kampasi et al. [124] and Wu et al. [122] (Figure 3a). Both probes contain silicon oxynitride waveguides (12 µm × 5 µm and 30 µm × 7µm) with silicon oxide (*n* = 1.46) cladding layers (2 µm and 3.5 µm) on top of silicon shanks with eight integrated recording electrodes. However, the method in which they try to improve the coupling efficiency of the external light source to the waveguide differs. While the first probe employs a gradient-index lens to focus the light, the second probe increases its overlap cross-sectional area by tapering the width of the waveguide to double its original size. However, the maximal overlap cross-sectional area for these types of waveguides is limited by the achievable thickness of the oxynitride layer. Fabricating thicker layers has proven difficult due to the inherently large tensile stresses in the dielectric layer and the extended plasma etching times. Nevertheless, low-stress, thicker waveguides have been achieved using polymers, such as SU-8 (*n* = 1.59), instead of oxynitride [87,123,125]. The probe presented by Schwaerzle et al. [123] contains four 15–20 µm by 12 µm (width and thickness) SU-8 waveguides, while Son et al. [87] presents a 20 µm by 15 µm SU-8 waveguide. The probe presented by Son et al. [87] also exhibits two other interesting properties (Figure 3b). Firstly, by implementing two levels of y-shaped optical splitters along the probe, they were able to provide multi-site illumination with a single-coupled light source. Secondly, they showed that the low-stress glass cladding layer below the waveguide (Figure 3b), can double as the top layer for incorporated microfluidic channels (Figure 3c) [125]. 

Light-delivery systems made with waveguides implemented on silicon neural probes clearly have their advantages. Firstly, probes can straightforwardly be scaled up to contain multiple electrode shafts for multi-site illumination [87,123]. Secondly, each shaft can contain both optical illumination and electrical recording, while maintaining the size of each shank below a width of 100 µm and thickness of 50 µm. However, this approach also has its downsides. Firstly, the small cross-sections of the waveguides can lead to high coupling losses [87], thus reducing the power efficiency of the system. Secondly, while the small cross-section of the probes are beneficial to reduce the FBR, the high-modulus silicon carriers (E ≈ 130–190 GPa [37]) that are employed can possibly still cause persistent irritation of the neural tissue as a result of micromotions [35,36]. Lastly, it should also be noted that the electrical signals recorded by these systems are prone to stimulation artifacts as a result of photovoltaic effects occurring during the high-intensity illumination of the incorporated recording electrodes [126,127]. However, to reduce this effect, designs including side-emitting waveguides, employing integrated micromirrors [128] or grating couplers [129], and shielded recording sites [54] have been presented.

#### 4.2.2. Optical Fibers

The second category of waveguide-based light-delivery system use optical fibers as their waveguide of choice. Optical fibers are, in comparison with the previous group of waveguides, pick-and-place components that are commercially available with a large range of different characteristics (e.g., NA, size, transmission spectrum, etc.). They can also easily be combined with all kinds of stand-alone electrodes (e.g., tetrodes, microwires, high-density probes, etc.), simplifying the manufacturing process for optrodes tremendously. Consequently many optrodes have used commercially available fibers in their experimental set-ups [45,127,130].

**Figure 4:**
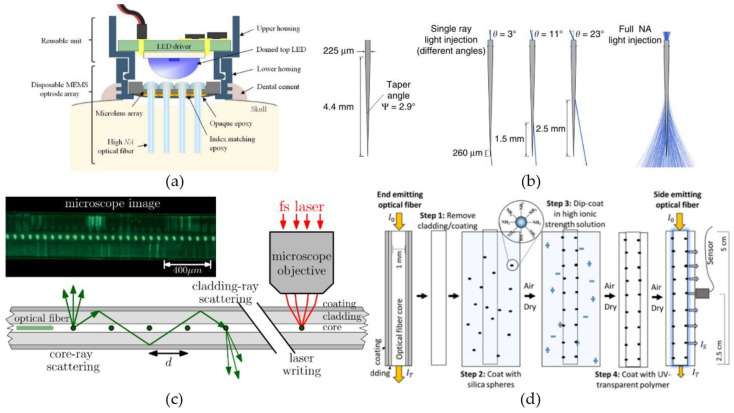
Multi-site and broad-range illumination with optical fibers. (**a**) A MEMS optrode array consisting out of a 4 × 4 array of optical fibers coupled to a single LED-source. Reproduced with permission from [130]; published by Elsevier, 2018. (**b**) Tapered optical fibers can both achieve light illumination at discrete positions along its length and across broad brain volumes, by injecting light at specific input angles and over the entire input range, respectively. Reproduced with permission from [132]; published by Springer Nature, 2017. (**c**) Modification of a step-index optical fiber by introducing femtosecond laser induced scattering centers, to obtain side emission at discrete positions along the length of the fiber. Reprinted with permission from [133] © The Optical Society. (**d**) Side-emitting optical fiber obtained by attaching nanoparticles, which function as scattering centers, on the surface of an uncladded glass fiber. Reprinted with permission from [134]. Copyright 2019 American Chemical Society.

Optical fiber systems can also provide multi-site and/or broad-range illumination. This can straightforwardly be implemented by incorporating multiple optical fibers into the design, whether or not coupled to the same integrated light source [130,131] (Figure 4a). An alternative, and less invasive way of obtaining multi-site and broad-range illumination with optical fibers, is by altering the geometrical properties of the fibers. For example, Pisanello et al. [132] presented that tapered fibers can be used to couple light out of the fibers at well-defined positions over a certain range (Figure 4b). The size of the range and the exact outcoupling position are defined by the NA and taper angle of the fiber, and the angle of light incoupling at the fiber interface, respectively. Additionally, homogenous light can be delivered to surrounding tissue, over a range of a few hundred µm to a few mm, by applying light to the fiber along all supported incoupling angles [126,132]. Another option to obtain tissue illumination at well-defined positions or over a certain range, is by introducing elements in the fibers to locally couple out the light (e.g., bends or microstructures). Reupert et al. [133] created side-emitting fibers by introducing femtosecond laser induced scattering centers along the fiber core (Figure 4c). The resulting local refractive-index fluctuations causes the transmitted light to scatter and couple out the fiber at the modified positions. Alternatively, nano-sized silica spheres attached to the surface of a glass optical fiber encapsulated in a transparent polymer (Figure 4d), has shown to produce similar light emission patterns [134]. The particles cause light to scatter by light refraction at the contact point between the silica spheres and the fiber core or by interactions of the particles with the light’s evanescent waves.

The aforementioned optical fibers fit well in a lot of optogenetic applications. Both their easy combinability with a large range of electrodes, as the possibility of customizing their illumination properties, make them a viable approach for the delivery of the opsin-enabling light. However, their relatively bulky size (diameter of 125–400 µm) and high young’s modulus (E_silica_ ≈ 80 GPa), make them less suitable for chronic implants and more for short-term optogenetic studies.

#### 4.2.3. Low-Modulus Waveguides and Optical Fibers

A wide range of low-modulus waveguides and optical fibers have been proposed from all kinds of biomaterials. These waveguides/fibers are discussed in the next few sections and are classified based on the type of biomaterial from which they are fabricated. There are three material groups that exhibit interesting properties for optical applications in biological environments, being the thermoplastics, the hydrogels and the elastomers. Table 2 contains a quick overview of some of the optical, mechanical and biological properties of the biomaterials and indicates for each group which of their properties are useful for optical components intended for optrode design. Further, for the waveguides and fibers discussed in the next few sections, the reported geometrical and optical characteristics are listed together with the manufacturing methods in Table 3.

##### Thermoplastic Polymers

**Figure 5:**
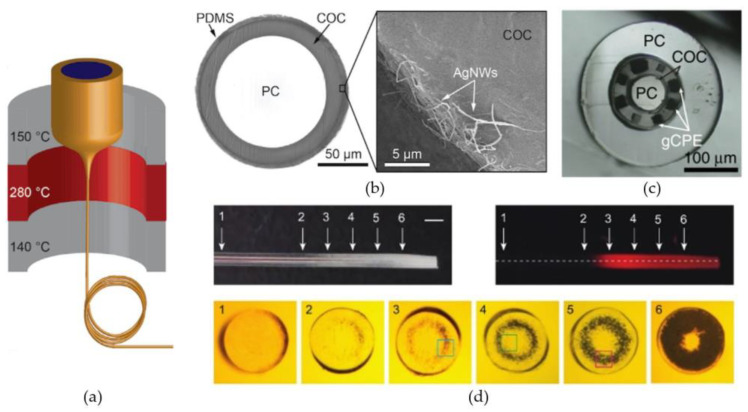
Optical fibers and fiber-based optrodes manufactured with thermoplastic polymers. (**a**) Thermal drawing of the PC/COC preform to obtain small optical fibers. (**b**) A cross-section of the PC/COC fiber after coating with AgNWs and PDMS. The SEM image depicts a close-up view of the AgNW electrode. Reproduced with permission from [137]; published by AAAS, 2017. (**c**) A cross-section of the multifunctional fiber presented by Park et al. [138], containing six incorporated graphite-conductive polyethylene (gCPE) electrodes and two microfluidic channels. Reproduced with permission from [138]; published by Springer Nature, 2017. (**d**) Photographs of the thermally drawn PES fiber after localized heat treatment. The amount of induced scattering centers is gradually increased from position 1 to position 6. For each position, the corresponding cross-section is given. The dark spots present in the cross-sections correspond to the induced microbubbles. Reprinted with permission from [142] © The Optical Society.

A first group of biomaterials, selected for their ability to create small-diameter, optical fibers, are the thermoplastic polymers. In this group, one of the most well-known materials for optical applications is poly(methylmethacrylate) or PMMA. Optical fibers created with PMMA, better known as polymer optical fibers or POFs, are widely used in short range, optical communication systems, due to their high mechanical durability and inexpensiveness in comparison to glass optical fibers [135]. Nevertheless, no optogenetic systems, which incorporate PMMA-based optical fibers, have been presented in literature. In contrast, optrodes have been presented with the thermoplastic polymers polycarbonate (PC) and cyclic olefin copolymer (COC) as core, respectively cladding material. Lu et al. [137] and Park et al. [138] proposed optrodes for which the optical fibers were created by a thermal drawing process. Thermal drawing is a fiber manufacturing process where a macroscale template or preform is heated to elevated temperatures and manually or automatically drawn into long fibers (Figure 5a). The obtained fibers can be up to hundreds of meters long and the cross-sections are downscaled versions of the original preform. The fiber proposed by Lu et al. [137] used a 12.5-mm-diameter preform composed out of COC sheets wrapped around a 9-mm-diameter, PC cylinder to draw a flexible, step-index fiber with a total diameter of 100–130 µm (Figure 5a). After the drawing process, silver nanowires (AgNWs) and an insulating PDMS layers were deposited on the surface of the fiber by two consecutive dip-coating steps (Figure 5b). The added layers increased the diameter of the fiber with only 5 µm, while allowing electrical recording of LFPs in the surrounding tissue. A similar optical fiber is proposed by Park et al. [138]. They present a multifunctional fiber for optogenetics, by adapting the previous design in two ways. Firstly, instead of adding the recording functionality after the drawing process, they incorporate six conductive-polymer electrodes in the preform (Figure 5c), providing multi-site electrical recording. Secondly, by also introducing two microfluidic channels, their design provides the possibility of delivering opsin carrying viral vectors or anti-inflammatory compounds to the surrounding neural tissue. Therefore, the total number of brain insertions can be reduced to one, since the implanted fiber can both function as transgene delivery system and as optogenetic neural probe.

Another thermally drawn fiber for optogenetics is presented by Shabahang et al. [142]. Here, they used polyethersulfone (PES) to fabricate flexible optical fibers with custom-designable scattering profiles (Figure 5d). Scattering losses along optical fibers can among other things be the result of surface roughness, impurities, compositional inhomogeneity or density fluctuations. Usually, these losses are minimized during processing to obtain transparent fibers with low optical transmission losses. However, homogenous light-outcoupling along the fiber or light-outcoupling at discrete positions and in discrete directions can be achieved, by controlling the scattering profile. Here, they employed the relatively high water absorption capacity (~2%) of the PES to create thermally-induced microbubbles. The microbubbles cause micrometer sized inhomogeneities along the fibers cross-section, that will scatter propagating light in all directions. Additionally, the amount of scattering at each position can be controlled by varying the heating profile and exposure time along the fiber. They demonstrated the viability of their process for fibers with a diameter down to 200 µm. Lastly, it should be mentioned that a range of optical fibers and waveguides have recently been presented made from poly lactic acid (PLA) and its derivatives [144,145,146,147]. However, these fibers are not suited for long-term optogenetic therapies, due to the fast degradation time of the material, which is in the order of weeks to months [147]. Therefore, these fibers will not be further discussed in this section, nor incorporated in Table 3.

##### Hydrogels

A second group of biomaterials that are worth examining are the hydrogels. These materials are synthetic or natural polymers that are extremely hydrophilic and swell when submerged in water. As a result, their Young’s modulus is relatively low and can obtain values similar to the one for neural tissue (Table 2).

The hydrogels that are most often employed to fabricate waveguides and optical fibers are made from alginate [31,155,156,159], polyethylene glycol (PEG) [150,159] polyacrylamide (PAAm) [31,156], and their derivatives [155]. Alginate hydrogel is a soft (E = 17 kPa [157]), natural material that exhibits low optical losses (≈1–2 dB/cm) as long as the hydrogel is created with low concentrations (1–2 wt%) of alginate. Consequently, the hydrogel has a refractive index (*n* = 1.3331 [155]) similar to the one of water (*n* = 1.33) and is therefore predominantly used as a cladding material [155,159]. Alginate is also often added to the synthetic PAAm-based hydrogels for the creation of large-strain enduring fibers. The alginate’s ionic crosslinks, intertwined between the PAAm’s covalent bonds, easily break when strain is applied and, therefore, absorb most part of the deforming energy [157]. Consequently, the stronger, covalent bonds in the alginate-PAAm hydrogel are spared and the resulting optical fiber can endure strains up to 700% [156]. Furthermore, the resulting fibers exhibit low optical losses and obtain Young’s moduli (E = 64–80 kPa) close to the modulus of neural tissue.

PEG-based hydrogels also have been proposed as suitable candidates for the development of mechanically soft optical fibers. There are two types of PEG that are generally used. Depending on the acrylate groups used in the monomers, the polymers are either called a PEG diacrylate (PEGDA) or a PEG dimethacrylate (PEGDMA). Hydrogels made from these materials have largely varying properties, depending on the underlying polymer network [148,150,172]. The refractive index, the Young’s Modulus and the swelling of PEG-based hydrogels have been reported with values ranging between 1.33 to 1.47, 60 kPa to tens of MPa’s and 1 to more than 20, respectively [148,150,172].

A PEG-based optical fiber was presented by Choi et al. [159]. They used PEGDA (700 Da) as the core material at 80–90 wt% in the precursor solution and employed an alginate cladding. As a consequence of using the short PEG monomers at high concentrations, causing a high crosslink density in the hydrogel matrix and low water intake, they obtained a relatively stiff (order of MPa) optical core with a high refractive index. However, the low-index alginate cladding makes for a soft mechanical buffer (E ≈ 20 kPa) with the surrounding tissue, while at the same time providing a large NA of 0.6. A similar fiber to the work of Choi et al. [159] was presented by Yetisen et al. [155]. Here, they introduced a PAAm copolymer to the PEGDA (700 Da) solution to increase the flexibility of the stiff PEG fiber, while still maintaining a NA of 0.54. Recently, also a poly(ethylene glycol dimethacrylate-co-N-isopropylacrylamide-co-acrylic acid) (PEGDMA-NIPAAm-AA) waveguide has been proposed, although the transmission losses were not reported [173]. Another innovation for PEG-based fibers was proposed by Ohannsmeier et al. [148]. They showed the viability of inserting polystyrene particles into the hydrogels to work as diffusors for controlled light-outcoupling.

A last thing that should be mentioned regarding hydrogel fibers, is the difficulty of manufacturing fibers with diameters equal or below 200–400 µm, similar to the size of standard glass fibers in fiber-based optrodes [45,127,130]. All of the discussed fibers use a similar manufacturing process. They UV-cure the hydrogels inside silicone [31,156] or PVC [155] tubes and then remove the fiber by air or water pressure, sometimes aided by swelling the silicone tube in dichloromethane [159]. However, this approach tends to limit the minimal obtainable diameter for step-index fibers to 300–400 µm (see Table 3), since the low mechanical strength and the high surface to volume ratio of the fibers, makes it increasingly difficult to remove the fibers from the tubes.

##### Elastomers

The third and last group of biomaterials discussed for the fabrication of waveguides and optical fibers are the elastomers. These materials are defined as synthetic polymers with rubber-like characteristics. Due to their rubber-like properties, elastomers usually have a low Young’s modulus (in the order of MPa) and can endure large strains before breaking. Lu et al. [137] fabricated an optical fiber from COC elastomer (COCE), a similar material to the earlier-mentioned thermoplastic COC, but with a Young’s modulus that is almost 100 times lower. The COCE is, similar to COC, qualified for thermal drawing, albeit with the aid of a sacrificial PMMA shell to help maintain its shape after drawing. The PMMA shell is removed after the COCE solidifies. Square fibers were created this way, with cross-sectional dimensions down to 100 µm. A second paper used citrate-based polymers to make a cylindrical, step-index fiber [161]. The fabrication process started by deposition of the liquid poly(octamethylene citrate) (POC) cladding material around a surface-polished stainless steel wire. After curing of the POC, the steel wire was removed and the cladding layer was used as the tube mold for the poly(octamethylene maleate citrate) (POMC) core. By using the surface-polished, steel-wire template, scattering losses due to surface roughness are reduced and very good control of the core diameter can be obtained. Unfortunately, the coating of the wires led to much less uniform layers and the eventual cladding layer was between 125 µm and 1.25 mm thick. Polyurethane (PU)) is a third type of elastomer used for optical fiber manufacturing. Zhao et al. [162] and Kwok et al. [163] presented a square and rectangular step-index fiber, respectively, with a core made of PU. While the material is interesting due to its relatively good optical transmission (>90% per cm), the cross-sectional area of the reported fibers is too large (7.2–9 mm^2^) to be employed in an optical-fiber based optrode.

Lastly PDMS has also proven itself to be a viable candidate for waveguide and optical fiber manufacturing. The material offers both good optical and mechanical properties, exhibits an excellent resistance against biodegradation and ageing, and is considered biocompatible [174,175]. Noteworthy, surface modification of the PDMS have shown to further improve the biocompatibility and to increase the cell viability at its surface by up to 650% [176,177], allowing for closer integration with the surrounding neural tissue. An example of a well-known PDMS is Sylgard 184, a room-temperature vulcanizing siloxane produced by Dow Corning. The optical transmission capabilities of Sylgard 184 (*n* = 1.40–1.41) optical fibers have been proven by Kwok et al. [165] and Wang et al. [166]. Both papers presented optical fibers with transmission losses lower than 1 dB/cm (see Table 3). They also demonstrated that PDMS is a suitable material to fabricate tapered waveguides [165] and has the ability to incorporate diffusors [166]. Further, step-index fibers and waveguides out of PDMS were also presented [167,170]. They used high-index elastomer (e.g OE-6550, LS-6257) with a Sylgard 184 cladding layer to obtain a large-angle-accepting structures (NA = 0.64–0.69) with low-loss transmission (0.14–0.36 dB/cm).

Sylgard 184 also has good mechanical properties, making it a widely used encapsulation material for implants [117,137,178]. The materials hydrophobic character and its low Young’s modulus (1–3 MPa) can protect internal electronic circuits from aqueous environment, while providing a soft interface with the surrounding tissue. Additionally, changing the curing temperature of the PDMS has shown to change the crosslink density, and therefore the Young’s modulus. For Sylgard 184, the modulus decreases from 2.97 MPa to 1.32 MPa by decreasing the curing temperature from 200 to 25 °C [168]. Similarly, the crosslink density can be reduced by altering the ratio of silicone precursor with crosslinking agent in the prepolymer solution. While a 10:1 ratio gives a modulus between 1 and 3 MPa, changing the ratio to 30:1, 50:1 and 60:1 reduces the modulus to approximately 100 kPa, 8 kPa and 3 kPa, respectively [169].

PDMS appears to be a viable material choice to fabricate waveguides with for deep-brain illumination. The biocompatible material provides a tunable modulus, low transmission losses and can be employed for homogenous or multi-site illumination (e.g., tapering, diffusors). Additionally, many fiber-manufacturing processes have been proposed, all with their own perks. Firstly, Martincek and co-authors [171], Li and co-authors [179], and Kacik and Martincek [180] all presented PDMS fibers manufactured with fiber drawing. This process allow for fibers with diameters ranging from a few mm down to several µm. Secondly, Snell et al. [181] showed an extrusion process capable of creating 200-µm-diameter fibers with a length in the order of tens of cm. Thirdly, the lost-wax method proposed by Lee and Kim [182] allows for the parallel manufacturing of many fibers with a highly-uniform cross-section and a diameter of only 150 µm.

## 5. Implantation Methods for Soft/Flexible Neural Implants

In the past few sections, both electrical and optical architectures were discussed with the goal of identifying suitable candidates to be combined in the design of a closed-loop optogenetic solution for long-term treatment of epilepsy. Flexible and/or small-cross-section optrodes are most promising in providing a long-term therapeutic intervention. Unfortunately, these probes usually do not exhibit sufficient mechanical stiffness required for penetrating the meningeal layers enveloping the brain and, therefore, attention to this practical problem has to be included in the design considerations of the optrode. Two main approaches exist for implantation of soft probes in the brain. The first approach is centered around implantation procedures (e.g., small surgical procedures and external guide systems). The second approach focuses on providing a temporarily increased stiffness by either manipulating the probe design or by temporarily implanting a stiff aid structure together with the probe.

### 5.1. External Measures for Eased Probe Implantation

A first widely used method to ease the mechanical requirements for implantation, is the surgical removal of the dura mater (the upper, most-dense meningeal layer) above the implantation site. This only leaves the weaker arachnoid mater and pia mater to be pierced by the probe. The surgical procedure is called a durotomy and can be combined with other methods to further reduce the required stiffness. One of those methods, is the collagenase-aided modification of the pia mater, proposed by Kralik et al. [183]. Collagenase partially breaks down the collagen network of the pia mater, making it easier to be penetrated. Kunal J. Paralikar and Ryan S. Clement [184] validated the method and showed that the collagenase mediated modification reduces the maximal insertion force, required to pierce the pia, up to 40%, hence, allowing thinner or more flexible probes to be implanted without buckling.

**Figure 6:**
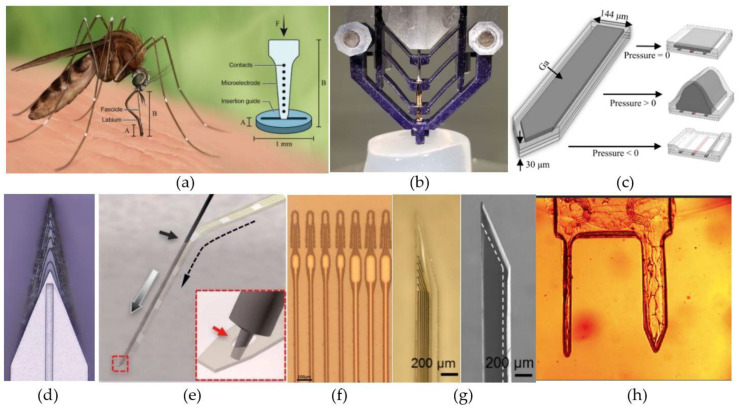
Methods to aid the implantation of flexible intracortical probes. (**a**) An external PMMA guide based on a mosquito’s labium. Reproduced with permission from [185]; published by Nature, 2018. (**b**) A microelectrode guide constructed out of four pairs of separated arms with a central opening through which the probe can pass. Reproduced with permission from [186]; published by IEEE, 2019. (**c**) A PDMS probe with an integrated microfluidic channel filled with gallium. Reproduced with permission from [187]; published by Elsevier, 2019. (**d**) A 3D-sharpened insertion shuttle with central wicking channel through which the dissolvable glue can flow and attach the shuttle to the probe. Reproduced with permission from [188]; published by IOP, 2019. (**e**) The principle behind the ‘needle-and-thread’ method. Reproduced with permission from [74]; published by AAAS, 2017. (**f**) Electrode array with integrated loops. Reproduced with permission from [189]; published by BioRxiv, 2019. (**g**) A phase contrast (**left**) and SEM (**right**) image from a parylene probe within a bilayered PVA/PLGA bioresorbable shuttle. The white dashed lines indicate the outer boundary of the parylene probe. Reproduced with permission from [97]; published by IOP, 2018. (**h**) A CMC dissolvable dual-shank needle. Reprinted with permission from ref. [73]. Copyright 2016 Springer Nature.

Other approaches have employed external guiding structures to increase the maximal force that can be applied to a probe during insertion without causing it to buckle, i.e., critical buckling force (CBF) [185,186]. Shoffstall et al. [185] presented a PMMA-based insertion guide inspired by the way that mosquitos use to penetrate the human skin (Figure 6a). By partially supporting the bottom of the probe, the guide is able to decrease the probe’s effective length, hereby, reducing the section of the probe that can buckle. As long as the length of the probe is less than five times the thickness of the guide, this method increases the CBF of small, flexible devices to more than four times its original value. Even larger improvements have been obtained with the external guide presented by Arafat et al. [186] (Figure 6b). They employed multiple pairs of supporting arms to further reduce the probe’s effective length and, hence, further increasing the CBF. For a 25-µm-diameter Pt microwire with a length of 16mm, the CBF increased from 254 µN to 5087 µN by employing the guide. By additionally spinning the probe during implantation, they were even capable of penetrating the dura mater.

### 5.2. Temporary Stiffeners

Several methods exist to increase the stiffness of an implantable components during insertion. Firstly, the probe itself can be designed to have a temporary stiffness during insertion while softening after implantation. Secondly, insertion shuttles that are implanted along with the probes, but are retracted afterwards, can be used. Thirdly, probes can be coated with stiff materials that dissolve upon probe implantation.

#### 5.2.1. Stiff Probes That Soften after Implantation

Stiff intracortical probes that soften after implantation would simplify the implantation procedure. A first way to obtain such probes consists of employing less conventional materials. Ware et al. presented softening intracortical electrodes made with a methyl acrylate (MA)/acrylic acid (AA) copolymer [190] and a thiol-ene/acrylate polymer [90]. Both materials originally have a large shear modulus of 700 MPa and 360–460 MPa, respectively, but soften due to plasticization by water uptake following implantation. The resulting shear moduli can go as low as 300 kPa and 4.7 MPa for the MA/AA copolymer and the thiol-ene/acrylate polymer, respectively. However, the water uptake that causes the probes to soften also causes the probes to swell. Ware et al. reported that the MA/AA copolymer probes could swell up to 200–300%, depending on the AA concentration, causing mechanical failure of the thin film metal electrodes, due to the resulting strain. In contrast, the thiol-ene/acrylate polymer only swells 3%, making it a more trustworthy technology.

Instead of using specialized materials, conventional, flexible intracortical probes (made out of PDMS, polyimide, parylene-C, etc.) can be equipped with a temporary stiffness by incorporating an embedded microfluidic channel (Figure 6c). By increasing the fluidic pressure in the embedded channel during implantation, the stiffness of the probe can be increased. Rezaei et al. [191] and Wen et al. [187] demonstrated probes that make use of this principle, and reported temporary improvements of the CBF with a factor five and ten, respectively. However, the main improvement reported by Wen et al. [187] came from their use of gallium inside the embedded microfluidic channel and as electrical interconnects for the electrodes (Figure 6c). The relatively stiff gallium (E = 10 GPa) has a melting temperature of approximately 30 °C and melts after the probe is implanted, hereby, reducing the overall stiffness with four orders of magnitude.

The discussed technologies are promising but they cannot be integrated with all types of probes, since they impose additional restriction during the design process. For example, intracortical implants created with MA/AA copolymer or thiol-ene/acrylate polymer require a minimal size to guarantee successful insertion. In addition, also the incorporation of microfluidic channels imposes a minimal size on the implant. For ultra-small probes, such as e.g., the NET or mesh electrodes, the following approaches are better suited.

#### 5.2.2. Insertion Shuttles

A second way to provide stiffness to soft, flexible probes involves the use of stiff carriers, such as silicon shuttles [188,192,193] or tungsten microwires [74,82,189,194], that are retracted upon probe implantation. The tricky part about this method is being able to detach the insertion shuttle after the probe has been delivered. Failing to do so can cause the probe to be displaced or fully explanted when the guide is retracted [193].

A first method to temporarily attach the stiff shuttle to the probe is by using a water-dissolvable glue (e.g., PEG). The U-shuttle presented by Du et al. [33] is ideally suited for fiber-based probes and fixes them in place by depositing a small amount of glue at the tip. In contrast, planar probes can more easily be implanted with accordingly flat guides [188,192] and often use integrated wicking channels (Figure 6d) for the glue to attach the shuttle at the backside of the probe. In addition, these shuttles can be sharpened, which reduces the required insertion force and, therefore, the required stiffness to penetrate the meningeal layers [188]. Further, the applicability of insertion shuttle arrays that allow the implantation of multiple probes in parallel, based on the temporary attachment with the dissolvable glue, has been demonstrated by Zhao et al. [194]. They presented multiple designs for minimally-invasive implantation of electrode arrays, to increase the overall throughput and allow for accurate control over the implantation sites.

A second method to temporarily attach shuttles to stiff carriers is by using a so called needle-and-thread approach [74,82,195] (Figure 6e,f). In this approach the probe functions as the needle and a stiff microwire [74] or even an actual needle [195] can function as the thread. For this method to work, the implantable probes have to incorporate a small hole at the probe tip. This hole is then used to lock the thread in place, after which the probe is pushed to the desired depth. Afterwards, the guide can easily be retracted, since it was never actually attached to the probe. Further, to allow for rapid and precise implantation of several electrodes, Hanson et al. recently reported the design of a computer controllable “sewing machine” [189].

Lastly, it should be mentioned that sometimes polymer-based materials, such as PI and PDMS, can exhibit hydrophobic interactions, which can cause the probes to stick to the insertion shuttles. To cope with this problem, Kozai et al. [193] demonstrated a carboxyl terminated self-assembled monolayer coating for the shuttles. The hydrophilic coating attracts water molecules to the insertion shuttle which causes it to detach more easily from the polymer probe.

#### 5.2.3. Dissolvable Coatings

A last way of providing temporary stiffness to small, flexible probes is by coating them with dissolvable, stiff materials (Figure 6g,h). A whole range of dissolvable materials have been used to insert probes. There are papers mentioning PEG [37,45,95,137,196], Silk fibroin [69,95,197], gelatin [72], poly-glycolic acid (PGA) [41], poly(lactic-co-glycolic) acid (PLGA) [97], poly(vinyl alcohol) (PVA) [97], carboxymethyl cellulose (CMC) [73] and maltose [92]. The Young’s modulus of these materials are relatively low (E ≤ 5 GPa). As a consequence, the required size of the dissolvable shuttle is generally larger than the size of its non-dissolvable counterpart. Consequently, these shuttles can be more invasive and can possible cause for an increase in the acute FBR. Nevertheless, depending on the used material, this is not always the case. Dissolvable needles made with gelatin, have shown to prevent scar tissue formation and to improve the neuronal density in the proximity of the probe, compared to the same probe implanted without gelatin [36,198].

Further, the dissolution rate of the guide is also an important factor to take into account when designing probes that employ dissolvable shuttles. Some materials, such as PLGA and PGA, have an intrinsically slow dissolution rate and take days to weeks to dissolve [41,97], while others, such as PEG, silk and PVA, can dissolve in the order of seconds or minutes [95,97]. Depending on the dissolution rate of the employed guide, more or less material will be required to guarantee a successful implantation. However, there are methods to decrease the effective dissolution rate. A first approach is by limiting the area that comes into contact with water. Takeuchi et al. [196] proposed a parylene-based microfluidic probe, which was stiffened by inserting PEG inside the microfluidic channel. Since the PEG had only a small contact area with the CSF, the dissolution went significantly slower and less material could be used to implant the probe. Secondly, the dissolvable materials, can also be coated with water retardation materials [72,97], which makes it harder for the CSF to reach the coated material.

## 6. Conclusions and Outlook

The design of an optogenetic probe for the closed-loop therapy of epilepsy and other related network disorders of the brain poses many challenges, of which most are related to providing and guaranteeing the long-term functionality in vivo. A first challenge is the preservation of the biological environment in which the optrode is present, to sustainably support recording of the epileptic networks and optical illumination of the genetically modified neurons, for the duration of treatment. However, the biological medium will always react to the optrode’s implantation and its continuous presence inside the medium, i.e., the FBR. This response includes persistent inflammation, glial scar formation, and neuronal loss in proximity of the implant, all of which counteract the implant’s functionality. Nevertheless, it has been shown that reducing an neural implant’s mechanical stiffness and overall size, can reduce the extent of the detrimental biological response to the point where optrodes can theoretically preserve their functionality indefinitely.

However, obeying both the stiffness and size restrictions, providing the optogenetic functionalities (electrical recording and optical illumination), as well as guaranteeing successful implantation has proven to be difficult and a plethora of designs have been presented in order to tray and solve this problem. Firstly, in the electrical domain, people have tried to circumvent the size and stiffness limitations by positioning the electrical contacts in regions which are less affected by the FBR. However, this showed only slight histological improvements compared with the traditional probes. Better results were obtained with the ultra-small and/or ultra-flexible designs, such as the NET-probes and mesh electrodes, which elicited (almost) no chronic biological reaction months past implantation. Additionally, to cope with the reduced SNR ratio, resulting from the smaller electrical contacts, most designs increased the effective surface area of the metal contacts by roughening the metal or creating micro-or nano protrusions. Secondly, in the optical domain, no clear solution for a long-term implantable light source, ideal for this application, has been reported yet. Both µLED- and waveguide-based approaches are capable of multi-site or broad-range illumination, but their functional longevity are limited by the device’s obtainable flexibility or size. More recently, less-conventional biomaterials have been used that are multiple orders of magnitude softer than the traditional biomaterials. The resulting low-modulus waveguides and optical fibers show promise in obtaining the desired specifications, although, further research has to be conducted. Lastly, also many approaches have been presented to solve the practical problem regarding the implantation of soft/flexible implants. These approaches include, but are not limited to, surgical procedures, external guiding systems and dissolvable guides. Furthermore, each method has its own strengths and weaknesses and multiple techniques can be combined. Nevertheless, the ideal implantation method will eventually depend on the electrical and optical component selected by the designer to constitute the optrode.

Finally, it should be noted that to the knowledge of the authors no of the discussed electrical or optical probes has been used for optogenetic closed-loop therapy of patients with epilepsy. However, this review may operate as a guide for future researchers and aid the design of an implant for human patients.

## Figures and Tables

**Table 1:** Impedance reduction by surface modification of metal contacts.

Metal Contact	Surface Modification	Surface Area (µm^2^)	Original Impedance ^1^ [kΩ]	Modified Impedance ^1^ [kΩ]	Ref.
Pt/Ir (90/10)	Pt particles	-	1520	11.96	[45]
Pt/Ir (90/10)	IrO_2_	-	2710	148	[45]
Au	Platinum black	177	-	207	[90]
Pt	Pt nanograss	962	1212 ± 365	413 ± 309	[27]
Pt	Pt nanograss	962	~300	~20	[91]
Au	Electrodeposited Au	100	2100	200–250	[89]
Au	CNT-Au nanocomposite	707	1090	59.02	[92]
Au	PEDOT	180	870 ± 330	46 ± 26	[82]
Au	PEDOT	707	329 ± 33	20.55 ± 0.82	[94]
Au	PEDOT	1257	630	~7	[95]
Au	PEDOT-PSS	154	-	41.5 ± 6.4	[97]
Ir	PEDOT-pTS	413	192.5 ± 10.0	35.0 ± 6.4	[93]
Au	PEDOT-CNT	707	329 ± 33	15.55 ± 0.67	[94]

^1^ All impedances are at the biological relevant frequency of 1 kHz. Abbreviations: CNT, carbon nanotube; PEDOT, poly(3,4-ethylenedioxythiophene); PSS, poly(styrene sulfonate); pTS, p-toluenesulfonate.

**Table 2:** Optical, mechanical and biological properties of biomaterials mentioned in Section 4.2.3.

Material	Refractive Index	Bulk Material Loss (λ = 473 nm) [dB/cm]	Young’s Modulus	Stability	Small-Cross-Section Fibers ^1^	References
Thermoplastic polymers	+	+/−	−	+	+	
PMMA	1.49	0.001	3 GPa	Inert	Yes	[135,136]
PC	1.58–1.586	1.27	2.39 GPa	Inert	Yes	[137,138,139]
COC	1.52–1.53	1.21–1.58	3 GPa	Inert	Yes	[137,138,140,141]
PES	1.65	2.6	2.6 GPa	Inert	Yes	[142,143]
PLA (including isomers and copolymers) ^2^	1.47	0.1–1.8	3.5 GPa	Degrades (weeks, months, 1–2 years)	No	[144,145,146,147]
Hydrogels	+	+/−	+	−	−	
PEG-based (PEGDA, PEGDMA, …)	1.33–1.46	0.13–1.26 ^3^	≥60 kPa	Slow oxidation and hydrolysis	No	[148,149,150,151,152,153,154]
PAAm	1.33–1.45	No data	8 kPa	Slow hydrolysis	No	[31,155,156,157,158]
Alginate	1.33	0.25–2.96 ^4^	17 kPa	Slow outdiffusion of divalent cations	/	[31,155,156,157,159,160]
Elastomer	+	+	+	+/−	+	
COCE	1.51	No data	34 MPa	Inert	Yes	[137]
POC/POMC	1.5	0.03–0.08	3.4–4.8 MPa	Degrades (months)	No	[161]
PU	1.49	0.02	4.7–7.4 MPa	Slow oxidation and hydrolysis	No	[162,163,164]
PDMS	1.40–1.55	0.11 (Sylgard 184)	3 kPa–10 MPa	Inert	Yes	[165,166,167,168,169]

^1^ Small-cross-section fibers is here defined as fibers with a diameter ≤200 µm. ^2^ Including poly(L-lactic acid), poly(D,L-lactic acid), poly(lactic-co-glycolic acid) and poly(D,L-lactic-co-glycolic acid). ^3^ Based on the reported transmission losses for PEGDA (700 Da) hydrogels at concentrations from 20–90% [150]. ^4^ Based on the reported transmission losses for Na alginate at concentrations from 1–4% [155]. Abbreviations: PMMA, poly(methylmethacrylate); PC, polycarbonate; COC, cyclic olefin copolymer; PES, polyethersulfone; PLA, poly lactic acid; PEG, poly-ethylene glycol; PEGDA, PEG diacrylate; PEGDMA, PEG dimethacrylate; PAAm, polyacrylamide; COCE, COC elastomer; POC, poly(octamethylene citrate); POMC, poly(octamethylene maleate citrate); PU, polyurethane; PDMS, polydimethylsiloxane

**Table 3:** Geometrical and optical properties, and manufacturing method of reported optical fibers.

Materials (Core/Clad)	Dimensions ^1^	Optical Loss [dB/cm]	λ [nm]	NA	Manufacturing Process	Ref.
PC/COC	100–130 µm ⌀ 65–71 µm ⌀	1.90 ± 0.02 <1.5	473 473	0.43 0.42	Thermal drawing Thermal drawing	[137,138]
PES	800 µm ⌀	0.7–0.8	633		Thermal drawing	[142]
Alginate-PAAm/Alginate-PAAm	750 µm/1100 µm ⌀	0.45	532	0.11	UV-curing in tube mold + dip-coating	[156]
Alginate-PAAm	300 µm ⌀ (unswollen)	0.249 (swollen)	472		UV-curing in tube mold	[31]
PEGDA (700Da)/Alginate	800 µm/1000 µm ⌀	0.32 ± 0.02	492	0.6	UV-curing in tube mold + dip-coating	[159]
P(AAm-co-PEGDA)/alginate	200 µm/300–400 µm ⌀	0.30	532	0.54	UV-curing in tube mold + dip-coating	[155]
COC elastomer	125 µm × 100 µm–250 µm × 200 µm	3.98	473		Thermal drawing	[137]
POMC/POC	500 µm/800 µm–3 mm ⌀	0.4	633	0.1	Thermal crosslinking in premanufactured cladding layer	[161]
PU/silicone	1 mm × 1 mm/3 mm × 3 mm	2	860	0.46	Thermal crosslinking in premanufactured cladding layer	[162]
PU/PDMS	4 mm × 1 mm/4 mm × 1.8 mm			0.48	Molding	[163]
PDMS	1 mm ⌀ 5 mm × 1.4 mm	0.63 0.45	441.6 445		Thermal crosslinking in tube mold	[166,165]
PDMS/PDMS	50 µm × 50 µm/ 250 µm × 100 µm	0.14	850	0.69	Soft lithography + capillary filling	[170]
PDMS/PDMS	800 µm/1100 µm ⌀	0.36 ± 0.03	635	0.64	Tube mold + dip-coating	[167]
PDMS	45 µm ⌀	0.5	632		Fiber drawing	[171]

^1^ Cylindrical fibers are indicated with “⌀” and the dimensions correspond with the diameter.
